# The Role of MicroRNAs in Diabetic Nephropathy

**DOI:** 10.1155/2014/920134

**Published:** 2014-09-01

**Authors:** Hao Wu, Lili Kong, Shanshan Zhou, Wenpeng Cui, Feng Xu, Manyu Luo, Xiangqi Li, Yi Tan, Lining Miao

**Affiliations:** ^1^Department of Nephrology, The Second Hospital of Jilin University, 218 Ziqiang Street, Changchun 130041, China; ^2^Chinese-American Research Institute for Diabetic Complications at Wenzhou Medical University, Wenzhou 325035, China; ^3^Kosair Children's Hospital Research Institute, Department of Pediatrics, University of Louisville, Louisville, KY 40202, USA; ^4^Cardiovascular Center, The First Hospital of Jilin University, Changchun 130021, China; ^5^State Key Laboratory of Molecular Biology, Institute of Biochemistry and Cell Biology, Shanghai Institutes for Biological Sciences, Shanghai 200031, China

## Abstract

Diabetic nephropathy (DN), as one of the chronic complications of diabetes, is the major cause of end-stage renal disease. However, the pathogenesis of this disease is not fully understood. In recent years, research on microRNAs (miRNAs) has become a hotspot because of their critical role in regulating posttranscriptional levels of protein-coding genes that may serve as key pathogenic factors in diseases. Several miRNAs were found to participate in the pathogenesis of DN, while others showed renal protective effects. Therefore, targeting miRNAs that are involved in DN may have a good prospect in the treatment of the disease. The aim of this review is to summarize DN-related miRNAs and provide potential targets for diagnostic strategies and therapeutic intervention.

## 1. Introduction

As one of the most important long-term complications of diabetes, diabetic nephropathy (DN) is the major cause of end-stage renal disease [1] and high mortality in diabetic patients. The main clinical features of DN are persistent albuminuria and progressively declined glomerular filtration rate (GFR). Microalbuminuria (30–300 mg a day of albumin in urine) indicates early DN while macroalbuminuria (>300 mg/day) represents DN progression [2]. The major pathological features of DN are characterized by hypertrophy and expansion in the glomerular mesangium and tubular compartments, along with podocyte dysfunction and accumulation of extracellular matrix (ECM) proteins. Several mechanisms, including hyperglycemia, advanced glycation end products, protein kinase C, oxidative stress, inflammation, and poly(ADP-ribose) polymerase activation, are believed to contribute to the pathogenesis and development of DN [3]. Several typical cell signaling pathways have been proven to be involved in DN. For example, transforming growth factor-*β* (TGF-*β*) is a well-known pathway leading to the accumulation of ECM in DN [4–6]. Phosphoinositide 3-kinase-protein kinase B (PI3K-Akt) pathway is considered to result in glomerular hypertrophy and ECM accumulation [7, 8]. Mitogen-activated protein kinase (MAPK) family including P38, extracellular signal-regulated kinases (ERK), and c-Jun N-terminal kinases pathways are also found to cause DN [9–13]. Nuclear factor kappa-light-chain-enhancer of activated B cells (NF-*κ*B), a key inflammatory pathway, recruits a variety of inflammatory cytokines involved in DN [14–16]. However, the molecular pathogenesis hidden behind is still not fully understood.

**Figure 1:**
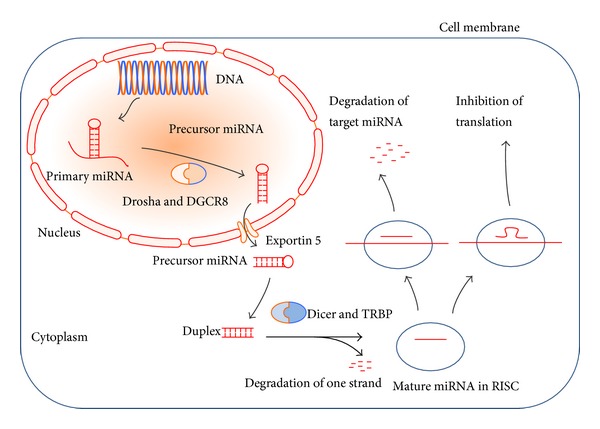
Biogenesis of miRNA. miRNAs are transcribed from DNA into primary-miRNAs (Pri-miRNAs) which contain hairpin-like structures. RNase III Drosha and its binding partner, DiGeorge syndrome critical region gene 8 (DGCR8), bind to the hairpin structures in Pri-miRNAs and process them into precursor miRNAs (Pre-miRNAs). Through Exportin 5, Pre-miRNAs are transferred into cytoplasm and are processed by another RNase III enzyme, Dicer, in collaboration with transactivating response RNA-binding protein (TRBP) to generate the mature miRNA duplex. One strand of the duplex goes into RNA-induced silencing complex (RISC), while the other is degraded. In RISC, mature miRNA recognizes target mRNAs through sequence complementarity, resulting in either degradation of the target mRNA (perfect complementarity to 3′UTR) or more frequently inhibition of translation (imperfect complementarity to 3′UTR).

MicroRNAs (miRNAs) are endogenously produced short noncoding RNAs of about 21–25 nucleotides that have been shown to play important roles in modulating gene expression, thus affecting almost every key cellular function [17, 18]. The biogenesis of miRNA has been largely understood and the canonical pathway was summarized in Figure 1. The final destiny of miRNAs is degradation in processing bodies [19–21]. It is estimated that about 60% of the human protein-coding genes can be targeted by miRNAs. Thus, research on miRNAs has attracted a high level of interest. Accumulating evidence has demonstrated that miRNAs are found to regulate signaling pathways involved in the pathogenesis of DN. For example, miR-192 targeted zinc finger E-box binding homeobox 1/2 (ZEB1/2) to activate TGF-*β* signaling pathway, leading to renal fibrosis proteinuria [22]. miR-21 targeted phosphatase and tensin homolog (PTEN) to induce the overactivation of Akt signaling pathway, followed by renal fibrosis and hypertrophy [23]. These DN-inducing miRNAs were found to be overexpressed in diabetic kidney, contributing to the pathogenesis of DN. In contrast, downregulated miRNAs showed renal-protective effects. Thus, we briefly summarize previous work by classifying the DN-related miRNAs into two groups, the upregulated (Table 1) and the downregulated (Table 2) classification of miRNAs, with the aim of providing a clear profile of DN-related miRNAs suggesting potential targets not only for diagnosis but also for therapeutic intervention.

## 2. Upregulated miRNAs in DN

Under diabetic conditions, several miRNAs are upregulated in diabetic kidney. These miRNAs bind to the 3′UTR of renoprotective genes which leads to their decreased expression. As a result, these upregulated miRNAs contribute to the pathogenesis of DN (Table 1).

### 2.1. miR-192

The pioneering work on miR-192 by Kato and coworkers indicated a central role of miR-192 in the development and progression of DN [22, 24, 25]. miR-192 was upregulated along with increased mRNA level of collagen 1 alpha 2 (COL1α2) compared with nondiabetic control in glomeruli isolated from streptozotocin- (STZ-) induced type 1 diabetic mice and db/db type 2 diabetic mice. Importantly, miR-192 was found to repress *δ*EF1 and Smad-interacting protein 1, which are repressors of COL1α1 and COL1α2 [24]. Another study showed miR-192-miR-200 cascade induced TGF-*β*1 expression. Thus, miRNA-regulated circuits may amplify TGF-*β*1 signaling, accelerating DN [22]. More recently, the same group found that TGF-*β* induced acetylation of chromatin and Ets-1 to alleviate repression of miR-192 in DN. The induction of miR-192 expression by TGF-*β* in mouse mesangial cells (MMCs) initially involved the Smad transcription factors, followed by sustained expression that was promoted by acetylation of the transcription factor Ets-1 and of histone H3 by the acetyltransferase p300 [25].

Putta et al. treated STZ-induced diabetic C57 mice with locked nucleic acid (LNA) modified anti-miR-192 and observed significantly increased ECM repressor ZEB1/2 and decreased expression of TGF-*β*, collagen, and fibronectin (FN) in diabetic kidney, as well as attenuated proteinuria [26], thus indicating the possibility of the approach of LNA-anti-miR-192 to the treatment of DN.

In contrast, Wang et al. found that TGF-*β* treatment decreased the expression of miR-192/215 in rat proximal tubular cells (NRK-52E), primary rat mesangial cells, human podocytes, and kidney of apolipoprotein E diabetic mice [27]. The discrepancies might be due to differences in cell types and animal species. It is impossible to confirm that these unconformities really exist under the same conditions. Further studies are needed to explain the differences between these results.

### 2.2. miR-216a and miR-217

Kato et al. dug out the miRNA-mediated link between TGF-*β* and Akt, which were important signaling pathways of DN in MMCs. miR-192 and TGF-*β* induced levels of MiR-216a and miR-217, both of which targeted PTEN, an inhibitor of Akt activation [28]. This work not only demonstrated the presence of miRNA-network regulated by miR-192/TGF-*β* but also, more importantly, indicated the mechanism of miRNA-mediated Akt activation by TGF-*β*. A further research showed that, under diabetic conditions, miR-216a was upregulated, followed by the inhibition of Y box binding protein 1 which led to increased expression of TGF-*β* stimulated clone 22, eventually resulting in high production of COL1α2 in MMCs [29]. This study suggested a fibrosis-inducing role of miR-216a related to the pathogenesis of DN in MMCs.

### 2.3. miR-200b/c

miR-200b and miR-200c are among the members of miR-200 family (miR-200a, miR-200b, miR-200c, and miR-141). miR-200b/c were found downstream of miR-192, and all three of them were able to induce TGF-*β*1, while miR-200b/c were both increased in glomeruli from type 1 (STZ) and type 2 (db/db) mice, as well as MMCs treated with TGF-*β*1, suggesting an miRNA-mediated positive feedback loop of TGF-*β*1 autoregulation in MMCs [22]. Besides, Park et al. observed a significant increase of miR-200b/c in diabetic mouse glomeruli and TGF-*β*-treated MMCs. TGF-*β* activated Akt in MMCs by inducing miR-200b and miR-200c, both of which targeted zinc finger protein Friend of GATA 2 (FOG2), an inhibitor of PI3K activation. Importantly, miR-200b/c inhibitors abrogated the TGF-*β*-induced increase in protein content to cell ratio. This study suggested a new mechanism for TGF-*β*-induced Akt activation through FOG2 downregulation by miR-200b/c, which led to glomerular mesangial hypertrophy in the progression of DN [30].

### 2.4. miR-21

Except for its critical role in tumorigenesis [31–33], miR-21 is also found as a DN player. miR-21 serves as the molecular link between high glucose and PTEN and contributes to renal cell hypertrophy and matrix expansion. Overexpression of miR-21 resulted in reduction in PTEN expression and increase in Akt phosphorylation, while miR-21 sponge, a miR-21 inhibitor, reversed the DN-inducing effects of high glucose. miR-21 also inactivated proline-rich Akt substrate of 40 kDa, a negative regulator of mammalian target of rapamycin complex 1 that can mediate pathologic features of DN [23]. In line with this study, work by Zhong et al. demonstrated miR-21 as a key therapeutic target for renal injury in db/db mice. The authors found miR-21 targeted mothers against decapentaplegic homolog 7 (SMAD7), which was the repressor of TGF-*β*1. Importantly, transferring miR-21 knockdown plasmids into the diabetic kidneys of db/db mice ameliorated microalbuminuria, renal fibrosis, and inflammation at age 20 weeks, revealing a therapeutic potential for DN by targeting miR-21 [34].

Fiorentino et al. found that, in a mice model of type 1 diabetes, SV40 MES 13 mouse mesangial cells, as well as human kidney biopsies from patients of DN, miR-21 were significantly upregulated, which led to downregulation of tissue inhibitors of metalloproteinase 3 (TIMP3) [35]. Given that TIMP3 deficiency has emerged as a hallmark of DN [36], it is conceivable that miR-21 may be an inducer of DN. In addition, work by Wang et al. in kk-ay type 2 diabetic mice demonstrated that miR-21 contributes to renal fibrosis by downregulating matrix metallopeptidase 9/TIMP1. The ECM inducing effect of miR-21 was reversed by antagomir-21 [37]. These two studies suggested that miR-21 induces DN through regulation of TIMPs.

### 2.5. miR-377

miR-377 was upregulated in high glucose cultured or TGF-*β* treated human and mouse mesangial cells. Increased miR-377 resulted in suppression of p21-activated kinase and superoxide dismutase, which enhanced FN expression [38]. To date, this has been the only study focused on the relationship between miR-377 and DN. Interestingly, another study showed that miR-377 targeted heme oxygenase 1 (HO-1), an important antioxidant which participated in oxidative redox signaling [39]. Since HO-1 also prevents DN through antioxidative effect [40, 41], miR-377/HO-1 pathway might be a new mechanism by which miR-377 induces DN. Further studies are needed to verify the underlying mechanisms.

### 2.6. miR-195

Elevated expression of miR-195 was found in both STZ-induced type 1 diabetic mice and podocytes cultured in high glucose. B-cell lymphoma 2 protein levels were decreased while caspase-3 increased in podocytes after transfection with miR-195 [42]. These findings suggested that miR-195 might mediate podocyte apoptosis in DN. In line with this study, miR-195 was observed to be increased not only in STZ-induced type 1 diabetic mice but also in high glucose cultured MMCs, followed by enhanced apoptosis of MMCs [43].

Besides, miR-195 was identified as an inhibitor of sirtuin 1 (Sirt1) in DN [44]. As a histone deacetylase, Sirt1 is a key regulator which ameliorates DN via multiple mechanisms [45–48]. It is interesting to investigate the regulation of Sirt1 by miR-195 in DN and inhibiting miR-195 might be a new strategy to ameliorate DN.

### 2.7. miR-215

Mu et al. identified miR-215 as an epithelial-mesenchymal transition-promoting molecule in TGF-*β*1 treated MMCs [49]. miR-215 was found to target catenin-beta interacting protein 1, which suppressed Wnt/*β*-catenin signaling. Thus miR-215 activated *β*-catenin followed by the overexpression of alpha smooth muscle actin (α-SMA) and FN.

### 2.8. miR-124

Podocytes are key components of the glomerular filtration barrier and adhere tightly to glomerular basement membrane (GBM) mainly through cell-matrix adhesion receptor INTEGRINα3*β*1 [50]. Li et al. found INTEGRINα3*β*1 as a target of miR-124 [51], indicating the possible role of miR-124 in podocyte adhesion damage under mechanical stress.

### 2.9. miR-1207-5p

Alvarez et al. reported that a long noncoding miRNA, miR-1207-5p, was highly expressed in normal human renal proximal tubule epithelial cells, podocytes, and normal mesangial cells and was upregulated by high glucose and TGF-*β*1; meanwhile miR-1207-5p also increased TGF-*β*1, PAI-1, and FN1, all of which contributed to DN [52].

### 2.10. miR-135a

He et al. showed that miR-135a was markedly upregulated in serum and renal tissue from patients with DN, as well as from db/db mice, accompanied by the development of microalbuminuria and renal fibrosis. Furthermore, the authors identified transient receptor potential cation channel, subfamily C, member 1 (TRPC1), as a target of miR-135a during renal injury. Overexpression of TRPC1 was able to reverse the pathological effects of miR-135a on promoting proliferation of mesangial cells and increasing synthesis of extracellular matrix proteins. Moreover, miR-135a attenuated store depletion-induced Ca (2+) entry into cells by regulating TRPC1. Importantly, knockdown of miR-135a in diabetic kidneys restored levels of TRPC1 and reduced synthesis of fibronectin and collagen 1* in vivo* [53]. These findings suggested that miR-135a plays an important role in renal fibrosis and inhibition of miR-135a might be an effective therapy for DN.

## 3. Downregulated miRNAs in DN

Several key factors are overexpressed in DN, such as TGF-*β*2, COL1, COL4, and NADPH oxidase subunit 4 (NOX4). These DN-inducing factors result in ECM accumulation, renal fibrosis, and oxidative stress, all of which contribute to the pathogenesis of DN. These DN-inducing factors are also targets of several miRNAs, which are downregulated in DN. It is reasonable that these downregulated miRNAs are DN-inhibiting miRNAs which lead to the decrease of these DN-inducing factors (Table 2).

### 3.1. miR-200a and miR-141

Although in the same family of miR-200, miR-200a and miR-141 seem to have opposite effects from miR-200b/c. In NRK52E cells, both TGF-*β*1 and -*β*2 downregulated miR-200a, which reduced expression of ECM proteins such as COL1, COL4, and FN, and so did miR-141. Interestingly, both miR-200a and miR-141 repressed TGF-*β*2 expression [54]. The study established a reciprocal inhibiting effect between miR-200a/miR-141 and TGF-*β*2. More recently, aldose reductase was found to elevate miR-200a-3p and miR-141 so as to coordinate kelch-like ECH-associated protein 1/NFE2-related factor 2, attenuating TGF-*β*1/2 signaling in both renal cortex of STZ-induced mice and MMCs [55].

### 3.2. miR-29

All three members of the miR-29 family (miR-29a/b/c) were suppressed by TGF-*β*1 in proximal tubular cells (NRK-52E), primary mouse mesangial cells, and human podocytes. miR-29 family repressed the expression of targeted COL1 and COL4 in both mRNA and protein levels [56]. In agreement with the study by Wang et al. [56], MiR-29a was downregulated in HK-2 cells (human proximal tubule cell line) under high glucose/TGF-*β*1 conditions. It directly targeted 3′UTR of COL4α1 and COL4α2, resulting in downregulation of these two fibrotic genes [57].

Study by Chen et al. demonstrated a renal-protective role of miR-29b in db/db mice, indicating that miR-29b may exert its protective effect by inhibiting TGF-*β*/SMAD3 signaling pathway and specificity protein 1/NF-*κ*B-driven renal inflammation [4]. A recent finding demonstrated that hyperglycemia-induced podocyte dysfunction was ameliorated by miR-29a promotion of nephrin acetylation [58].

Different from findings aforementioned, by using a miRNA expression array, Long et al. found miR-29c as an important miRNA in inducing cell apoptosis and accumulation of ECM under diabetic environment. The authors also identified Sprouty homolog 1 as a direct target of miR-29c. Albuminuria and kidney ECM were reduced by knockdown of miR-29c with antisense oligonucleotide in db/db mice [59]. The discrepancies may be due to differences in experimental models. Further studies are required to confirm the controversial results.

### 3.3. miR-451

To date, only one study has shown the DN-preventing role of miR-451. Zhang et al. defined tyrosine 3-monooxygenase/tryptophan 5-monooxygenase activation protein, zeta (YWHAZ), as a target of miR-451 and overexpression of miR-451 caused reduction of p38-MAPK signaling via suppression of YWHAZ [60], revealing the potential therapeutic role of miR-451 since p38-MAPK pathway was positively involved in DN [10]. However, in cancer research, a variety of studies have shown the tumor suppressing effect of miR-451. miR-451 targeted 14-3-3zeta, a phosphoserine/threonine-binding protein that inhibited nuclear accumulation of transcription factor FoxO3, a positive regulator of erythroid antioxidant genes [61]. miR-451 also inhibited cell proliferation in human hepatocellular carcinoma through direct suppression of inhibitor of kappa B kinase-beta, leading to the downregulation of NF-*κ*B [62]. Zhang et al. also validated that miR-451 targeted CUG triplet repeat-binding protein 2, a ubiquitously expressed RNA-binding protein, known to interact with cyclooxygenase-2 (COX-2) 3′UTR and inhibit its translation [63]. Since each of FoxO3, NF-*κ*B, and COX-2 plays a role in DN [15, 64, 65], it is possible that miR-451 may ameliorate DN through regulating these factors. Further studies are needed to verify the hypothesis.

### 3.4. miR-25

miR-25 level was significantly reduced both in kidneys from diabetic rats and in high glucose-treated mesangial cells, accompanied by the increases in NOX4 expression levels. An inhibitor of miR-25 effectively increased NOX4 levels. Luciferase assays showed that miR-25 directly bound to the 3′UTR of NOX4 mRNA. These data indicate that miR-25 may be a DN-protective molecule through inhibiting NOX4 [66].

### 3.5. miR-93

Long et al. identified vascular endothelial growth factor A (VEGF-A) as a putative target of miR-93 in kidneys of db/db mice. Cell experiments showed the forced expression of miR-93 abrogated VEGF protein secretion, while miR-93 inhibitors increased the secretion of VEGF [67].

### 3.6. let-7b

TGF-*β*1 decreased let-7b expression and induced fibrogenesis in NRK52E cells while ectopic expression of let-7b inhibited TGF-*β*1 receptor 1 (TGFBR1) expression, leading to reduced expression of ECM genes. Conversely, knockdown of let-7b elevated TGFBR1 expression and mimicked the profibrotic effect of TGF-*β*1. Importantly, let-7b expression was also reduced in kidneys of type 1 diabetic mice together with upregulated TGFBR1 [68]. Thus, let-7b seemed to show a good prospect for therapeutic intervention of renal fibrosis in DN. However, work by Schaeffer et al. [69] showed elevated let-7b under high glucose conditions, the result of which was reduced expression of transcription factor high-mobility group AT-hook 2, in turn reducing laminin subunit beta-2, which was regarded as a key component of GBM and determined glomerular barrier permeability. Further studies are needed to confirm the exact role of let-7b in DN.

## 4. Therapeutic Speculation of miRNAs in DN

Because of the important role of miRNAs in regulating multiple biological effects in DN, it is of great potential to develop methods to inhibit DN-inducing miRNAs or increase kidney-protective miRNAs. The aforementioned upregulated or downregulated miRNAs may be potential targets for the treatment of DN.

### 4.1. Silencing DN-Inducing miRNAs

There are basically four ways to silence miRNAs, including anti-miRNA oligonucleotides (AMOs), miRNA-inhibiting natural agents, miRNA sponges, and gene knockout [70]. All four methods are briefly introduced below.

#### 4.1.1. Anti-miRNA Oligonucleotides (AMOs)

AMOs are designed to complement miRNAs that are stopped from binding to their target sequences [71]. However, delivery of AMOs* in vivo* is a substantial obstacle to their effective use as therapeutics. Chemical modification of AMOs can be beneficial by improving hybridization affinity for the target mRNA, resistance to nuclease degradation, or activation of RNaseH or other proteins involved in the terminating mechanism [72]. 2′-O-Me modification as well as the 2′-O-methoxyethyl (2′-MOE) and 2′-fluoro (2′-F) chemistries is modified at the 2′ position of the sugar moiety, while LNA comprises a group of bicyclic RNA analogues in which the furanose ring in the sugar-phosphate backbone is chemically locked in an RNA mimicking N-type (C3′-endo) conformation by the introduction of a 2′-O,4′-C methylene bridge [72–76]. Among these chemical modifying methods, LNA shows the highest affinity towards complementary RNA [77, 78].

Inhibiting DN-inducing miRNAs with AMOs represented a good profile in the treatment of the disease. Transfer of miR-21 knockdown plasmids which contained LNA-anti-miR-21 into the diabetic kidneys of db/db mice at age 10 weeks significantly attenuated microalbuminuria, renal fibrosis, and inflammation at age 20 weeks [34]. Multiple low dose administration of LNA-anti-miR-192 in type 1 diabetic mice resulted in decreased miR-192 level, together with decreased COL1α2 and miR-216a/miR-217 and attenuated Akt activation [28]. In another study, injection with LNA-anti-miR-192 decreased the expression of miR-192, miR-141, miR-200b, miR-200c, COL1α2, COL4α1, and TGF-*β*1 in mouse renal cortical tissues [22]. Similarly, LNA-anti-miR-192 ameliorated DN in C57 type 1 diabetic mice by restoring the function of ECM inhibitor ZEB1/2, leading to downregulation of ECM genes and less albuminuria [26]. Knockdown of miR-29c by a specific antisense oligonucleotide significantly reduced albuminuria and ECM in kidneys of db/db mice [59]. miR-215 silencing* in vivo* with antagomir-215 significantly reduced miR-215-mediated *β*-catenin activity and decreased a-SMA and FN expression in db/db mice [49]. Antagomir-21 decreased TIMP1, COL4, and FN proteins as well as urine albumin creatinine ratio (ACR) and creatinine clearance ratio (CCR) in kk-ay mice [37]. These findings suggested a potential therapeutic prospect of AMOs in clinical use.

#### 4.1.2. miRNA-Inhibiting Natural Agents

Some natural agents derived from food are demonstrated to have miRNA-inhibiting effect. Curcumin and its analog CDF were found to downregulate miR-21, a key miRNA in tumor aggressiveness [79]. Resveratrol also reduced prostate cancer growth and metastasis by inhibiting Akt/miR-21 pathway [80]. Because miR-21 contributes to DN, it is quite possible that curcumin and resveratrol may ameliorate DN through inhibiting miR-21 [7, 34, 36, 37].

#### 4.1.3. miRNA Sponges

miRNA sponges contain complementary binding sites to the seed region of the miRNA of interest, which allows them to block a whole family of related miRNAs [70]. The sponges are transferred into cells by subcloning the miRNA binding site region into a vector containing a U6 small nuclear RNA promoter with 50 and 30 stem-loop elements [81].

A study using miR-21 sponge effectively inhibited endogenous miR-21 at the cellular level and prevented downregulation of PTEN and phosphorylation of Akt induced by high glucose in rat and human mesangial cells [23]. However, the application of miRNA sponges in DN animal models has not been reported, although it has already been used in animal models of cardiac hypertrophy, immune response, and breast cancer [82–84]. Studies in animal models are needed to observe the utility of this method towards DN.

#### 4.1.4. Genetic Knockout

Gene-knockout mice lose the whole function of miRNAs. The knockouts can be either a miRNA itself or key miRNA processing factors such as Drosha, Dicer, and argonaute 2 (Ago2). Mice with podocyte-specific deletion of Dicer induced proteinuria and glomerulosclerosis [85]. Another study showed that Dicer-knockout in podocytes led to rapid glomerular and tubular injury [86].

On the other hand, a specific miRNA knockout showed a promising effect on the prevention of DN. miR-192-knockout mice were protected from key features of DN [87] and miR-21-knockout mice suffered far less interstitial fibrosis in response to kidney injury [88].

### 4.2. Restoring Renal-Protective miRNAs

By using miRNA mimics, miRNA expression vectors, miRNA-containing exosomes, and miRNA-inducing natural agents, levels of renal-protective miRNAs can be restored and thus lead to the protection from DN.

#### 4.2.1. miRNA Mimics

miRNA mimics are double-stranded synthetic miRNA oligonucleotides. The guide strand is identical to the mature miRNA sequence, while the other strand called passenger strand is partially or fully complementary to the guide strand [89]. When transfected into cells, the guide strand which mimics the function of certain miRNA regulates protein-coding genes in a miRNA-like manner [90]. miRNA oligo mimics are easy to synthesize and introduce into cells using lipid reagents or electroporation and are easily achievable in most cellular situations. However, high cost for synthesis and purification and rapid clearance following transfection are the disadvantages. A variety of miRNA mimics showed miRNA mimicking effects on cancers both* in vitro* and* in vivo* [91–94]. However, there is no study focused on miRNA mimics in DN. Thus, replacement of renal-protective miRNAs would be a new strategy for the attempt to the treatment of DN.

#### 4.2.2. miRNA Expression Vectors

miRNA expression vectors are engineered to express miRNAs of interest. In a plasmid or viral vector, a certain miRNA can be expressed by a short hairpin RNA (shRNA) using polymerase II or III promoter. The shRNA is processed into mature miRNA by Dicer before loading into RISC [95]. Artificial miRNA vector which contained natural miRNA precursor inhibited the expression of target mRNA [96]. The knockdown effect of shRNAs on the expression of target gene was striking and stable [74]; however, it might saturate the Exportin 5 pathway of endogenous miRNAs, leading to off-target effects with fatal consequences [97]. miRNA expression vectors have the advantages of longer expression and reduced likelihood of off-target effects because the guide and passenger strands are completely natural to the cell [89]. The limitations in clinical applications include possible insertion of genetic material into the specific location of the genomes of the host cells and causing possible immune responses. To date, no studies utilizing miRNA vectors are found on DN.

#### 4.2.3. miRNA-Containing Exosomes

Exosomes are 40–100 nm membrane vesicles which contain proteins, mRNAs, miRNAs, or signaling molecules and are secreted into the extracellular space by numerous cell types [98]. Valadi et al. demonstrated that exosomes transferred miRNAs from their cell of origin to target cells [99]. In addition to miRNAs, pre-miRNA could be identified in mesenchymal stem cell-derived exosomes [100]. Therefore, the miRNAs-transferring ability of exosomes offers the promise that they may be used for therapeutic purposes for DN. Exosomal miRNAs have been discovered as diagnostic biomarkers of DN [101, 102]. However, studies on exosomal miRNAs in preventing or ameliorating DN are still wanted in the future.

#### 4.2.4. miRNA-Inducing Natural Agents

Difluorinated curcumin (CDF), a curcumin analog, increased the expression of miR-200a in pancreatic cancer cells [103]. Isoflavone and 3,3′-diindolylmethane (DIM) restored the expression of let7-b and led to the reversal of epithelial-to-mesenchymal transition in gemcitabine-resistant pancreatic cancer cells [104]. Because of the renal-protective role of miR-200a [54, 105] and let7-b [68], CDF, isoflavone, and DIM might prevent DN through regulating these two miRNAs.

To date, there has not been a successful clinical intervention of miRNAs towards diseases. However, several miRNA interventions have been in different clinical developmental stages. miR-122 inhibitor against hepatitis C virus infection is in phase II clinical trial [106]. miR-34 mimic against liver cancer or metastasized cancer involving liver is in phase I clinical trial [90]. let-7 mimic against cancer (details undisclosed) is in preclinical stage [107]. Inhibitors of miR-21, miR-208, miR-195, miR-221, miR-103/105, and miR-10b are in preclinical stage against cancer/fibrosis, heart failure/cardiometabolic disease, postmyocardial infarction remodeling, hepatocellular carcinoma, insulin resistance, and glioblastoma, respectively [107]. It is noted that, among these miRNAs, miR-21 and miR-195, let-7 are all DN-related, which might provide hope for the treatment of DN.

## 5. Conclusions

In summary, several miRNAs are related to DN. Some of them take part in the pathogenesis and development of the disease while others serve as DN-killers or -preventers. Therefore, it would be wise to elevate the renal-protective miRNAs and reduce DN-inducing ones.

miRNAs established a vast ocean for researchers to dive into and find the pathogenesis of disease and the potential target for therapeutic intervention. The prospect of miRNA-based intervention is bright. However, there are still challenges. For example, the exact and detailed regulation and function of miRNAs are still not fully understood. A certain miRNA may have several target genes. Thus, either upregulation or downregulation of a miRNA would have multifactorial effects, including the expected effect as well as side effects. Experimental verification of target genes also seems to be hard work, for the miRNA regulations are basically at a translation level. Better understanding of miRNA biogenesis and function will be beneficial for better application of miRNA-based treatment for diseases, including DN.

## Figures and Tables

**Table 1:** Upregulated miRNAs.

miRNAs	Targets	Biological outcome	Models	References
miR-192	*δ*EF1, SIP1	COL1*α*1 and COL1α2 ↑	Diabetic mice (STZ), db/db mice	[24]
	ZEB1/2	TGF-*β*, Col, FN ↑, proteinuria ↓	Diabetic mice (STZ)	[22]

miR-216a	PTEN, YBX1	MMC survival, hypertrophy, COL1*α*2 ↑	MMCs	[28, 29]

miR-217	PTEN	MMC survival, hypertrophy	MMCs	[28]

miR-200b/c	ZEB1	TGF-*β*1, COL1*α*2, COL4*α*1 ↑	Diabetic mice (STZ), db/db mice, MMCs	[22]
	FOG	p-Akt, ERK ↑, hypertrophy	Diabetic mice (STZ), MMCs	[30]

miR-21	PTEN, PRAS40	p-Akt, mTORC1, hypertrophy, COL1*α*2, FN ↑	HMCs	[23]
	SMAD7	Microalbuminuria, TGF-*β*, NF-*κ*B ↑	db/db mice	[34]
	TIMP3	TIMP3 ↓	Diabetic mice (STZ), MMCs, kidney biopsy (human)	[35]
	TIMP1	COL4, FN, ACR ↑; CCR ↓	kk-ay mice	[37]

miR-377	PAK1, SOD	FN ↑	Diabetic mice (STZ), MMCs, HMCs	[38]

miR-195	BCL2	Caspase-3, caspase-8 ↑	Diabetic mice (STZ), podocytes, MMCs	[42, 43]

miR-215	CTNNBIP1	*β*-Catenin, FN, α-SMA ↑		[49]

miR-124	INTEGRIN*α*3*β*1	Urinary podocyte nephrin, podocin, albumin ↑	Diabetic rats (STZ)	[51]

miR-29c	SPRY1	Albuminuria, ECM ↑	db/db mice	[59]

miR-1207-5p		TGF-*β*1, PAI-1, FN ↑	HK-2 cells, podocytes, normal mesangial cells	[52]

miR-135a	TRPC1	Microalbuminuria ↑, renal fibrosis ↑	db/db mice	[53]

STZ: streptozotocin; *δ*EF1: deltaEF1 (ZEB1); FOG: Friend of GATA; SIP1: Smad-interacting protein 1; Col: collagen; ZEB1/2: zinc finger E-box binding homeobox 1/2; YBX1: Y box binding protein 1; α-SMA: alpha smooth muscle actin; PTEN: phosphatase and tensin homolog; p-Akt: phosphorylated protein kinase B; PRAS40: proline-rich Akt substrate 40; mTORC1: mechanistic target of rapamycin complex 1; SMAD3: mothers against decapentaplegic homolog 3; SMAD7: mothers against decapentaplegic homolog 7; TIMP: tissue inhibitors of metalloproteinase; PAK1: p21 activated kinase; SOD: superoxide dismutase; BCL2: B-cell CLL/lymphoma 2; INTEGRIN*α*3*β*1: integrin alpha 3 beta 1; SPRY1: Sprouty homolog 1; NF-*κ*B: nuclear factor kappa B; TGF-*β*: transforming growth factor beta; ERK: extracellular signal-regulated kinases; ECM: extracellular matrix; FN: fibronectin; PAI-1: plasminogen activator inhibitor-1; MMC: mouse mesangial cell; HMC: human mesangial cell; RMC: rat mesangial cell; ACR: albumin creatinine ratio; CCR: creatinine clearance ratio; TRPC1: transient receptor potential cation channel, subfamily C, member 1.

**Table 2:** Downregulated miRNAs.

miRNAs	Targets	Biological outcome	Models	References
miR-200a/miR-141	TGF-*β*2	COL1, COL4, FN ↓	NRK52E cells	[54]

miR-29a/b/c	COL1, COL4	COL1, COL4 ↓	NRK52E cells, MMCs, human podocytes	[56]

miR-29a	COL4*α*1/2 HDAC4	COL1, COL4 ↓ Podocytes dysfunction ↓	HK-2 cells miR-29a transgenic mice Podocytes	[57] [58]

miR-29b		TGF-*β*/SMAD3, Sp1/NF-*κ*B ↓	db/db mice	[4]

miR-451	YWHAZ	p38MAPK, ECM ↓	MMCs	[60]

miR-25	NOX4	NOX4 ↓	RMCs	[66]

miR-93	VEGF-A	VEGF, COL4*α*3, FN ↓	db/db mice, podocytes, renal microvascular endothelial cells	[67]

Let-7b	TGFBR1	SMAD3, ECM ↓	Diabetic mice (STZ), NRK52E cells	[68]

TGFBR1: transforming growth factor beta receptor 1; VEGF-A: vascular endothelial growth factor A; Sp1: specificity protein 1; HDAC4: histone deacetylase 4; YWHAZ: tyrosine 3 monooxygenase/tryptophan 5-monooxygenase activation protein, zeta; NOX4: NADPH oxidase subunit 4; NRK52E cells: rat renal proximal tubular cell line.

## Abbreviations

ACR: Albumin creatinine ratio
AMOs: Anti-miRNA oligonucleotides
α-SMA: Alpha smooth muscle actin
BCL2: B-cell CLL/lymphoma 2
CCR: Creatinine clearance ratio
Col: Collagen
DN: Diabetic nephropathy
ECM: Extracellular matrix
ERK: Extracellular signal-regulated kinases
FN: Fibronectin
FOG: Friend of GATA
HDAC4: Histone deacetylase 4
HMC: Human mesangial cell
MAPK: Mitogen-activated protein kinase
miRNA: MicroRNA
MMC: Mouse mesangial cell
mTORC1: Mechanistic target of rapamycin complex 1
NF-*κ*B: Nuclear factor kappa B
NOX4: NADPH oxidase subunit 4
NRK52E: Rat renal proximal tubular cell line
PAI-1: Plasminogen activator inhibitor-1
PAK1: p21 activated kinase
PI3K-Akt: Phosphoinositide 3-kinase-protein kinase B
PRAS40: Proline-rich Akt substrate 40
RISC: RNA-induced silencing complex
RMC: Rat mesangial cell
SOD: Superoxide dismutase
Sp1: Specificity protein 1
SPRY1: Sprouty homolog 1
STZ: Streptozotocin
TIMP: Tissue inhibitors of metalloproteinase
TGF-*β*: Transforming growth factor-*β*
TGFBR1: Transforming growth factor beta receptor 1
TRPC1: Transient receptor potential cation channel, subfamily C, member 1
VEGF-A: Vascular endothelial growth factor A
YBX1: Y box binding protein 1
YWHAZ: Tyrosine 3 monooxygenase/tryptophan 5-monooxygenase activation protein, zeta
ZEB1/2: Zinc finger E-box binding homeobox 1/2.

## References

[1] Dronavalli S, Duka I, Bakris GL (2008). The pathogenesis of diabetic nephropathy. *Nature Clinical Practice Endocrinology and Metabolism*.

[2] Alvarez ML, DiStefano JK (2013). The role of non-coding RNAs in diabetic nephropathy: potential applications as biomarkers for disease development and progression. *Diabetes Research and Clinical Practice*.

[3] Sun YM, Su Y, Li J, Wang L (2013). Recent advances in understanding the biochemical and molecular mechanism of diabetic nephropathy. *Biochemical and Biophysical Research Communications*.

[4] Chen HY, Zhong X, Huang X (2013). MicroRNA-29b inhibits diabetic nephropathy in db/db mice. *Molecular Therapy*.

[5] Reeves WB, Andreoli TE (2000). Transforming growth factor *β* contributes to progressive diabetic nephropathy. *Proceedings of the National Academy of Sciences of the United States of America*.

[6] Ziyadeh FN, Sharma K (2003). Overview: combating diabetic nephropathy. *Journal of the American Society of Nephrology*.

[7] Dey N, Ghosh-Choudhury N, Kasinath BS, Choudhury GG (2012). TGF*β*-stimulated microRNA-21 utilizes PTEN to orchestrate AKT/mTORC1 signaling for mesangial cell hypertrophy and matrix expansion. *PLoS ONE*.

[8] Habib SL, Yadav M, Tizani S, Bhandari B, Valente AJ (2012). Tuberin inhibits production of the matrix protein fibronectin in diabetes. *Journal of the American Society of Nephrology*.

[9] Adhikary L, Chow F, Nikolic-Paterson DJ (2004). Abnormal p38 mitogen-activated protein kinase signalling in human and experimental diabetic nephropathy. *Diabetologia*.

[10] Sakai N, Wada T, Furuichi K (2005). Involvement of extracellular signal-regulated kinase and p38 in human diabetic nephropathy. *The American Journal of Kidney Diseases*.

[11] Lin CL, Wang F, Kuo Y, Huang Y, Huang H, Sun Y (2006). Ras modulation of superoxide activates ERK-dependent fibronectin expression in diabetes-induced renal injuries. *Kidney International*.

[12] Zhang L, Pang S, Deng B (2012). High glucose induces renal mesangial cell proliferation and fibronectin expression through JNK/NF-*κ*B/NADPH oxidase/ROS pathway, which is inhibited by resveratrol. *International Journal of Biochemistry and Cell Biology*.

[13] Pan Y, Zhang X, Wang Y (2013). Targeting JNK by a new curcumin analog to inhibit NF-kB-mediated expression of cell adhesion molecules attenuates renal macrophage infiltration and injury in diabetic mice. *PLoS ONE*.

[14] Bhattacharya S, Manna P, Gachhui R, Sil PC (2013). D-Saccharic acid 1,4-lactone protects diabetic rat kidney by ameliorating hyperglycemia-mediated oxidative stress and renal inflammatory cytokines via NF-*κ*B and PKC signaling. *Toxicology and Applied Pharmacology*.

[15] Ka SM, Yeh YC, Huang XR (2012). Kidney-targeting Smad7 gene transfer inhibits renal TGF-*β*/MAD homologue (SMAD) and nuclear factor *κ*b (NF-*κ*B) signalling pathways, and improves diabetic nephropathy in mice. *Diabetologia*.

[16] Xie X, Peng J, Chang X (2013). Activation of RhoA/ROCK regulates NF-*κ*B signaling pathway in experimental diabetic nephropathy. *Molecular and Cellular Endocrinology*.

[17] Bhatt K, Mi QS, Dong Z (2011). MicroRNAs in kidneys: biogenesis, regulation, and pathophysiological roles. *American Journal of Physiology—Renal Physiology*.

[18] Fernandez-Valverde SL, Taft RJ, Mattick JS (2011). MicroRNAs in *β*-cell biology, insulin resistance, diabetes and its complications. *Diabetes*.

[19] Chua JH, Armugam A, Jeyaseelan K (2009). MicroRNAs: biogenesis, function and applications. *Current Opinion in Molecular Therapeutics*.

[20] Kim VN, Han J, Siomi MC (2009). Biogenesis of small RNAs in animals. *Nature Reviews Molecular Cell Biology*.

[21] Zamore PD, Haley B (2005). Ribo-gnome: the big world of small RNAs. *Science*.

[22] Kato M, Arce L, Wang M, Putta S, Lanting L, Natarajan R (2011). A microRNA circuit mediates transforming growth factor-*β*1 autoregulation in renal glomerular mesangial cells. *Kidney International*.

[23] Dey N, Das F, Mariappan MM (2011). MicroRNA-21 orchestrates high glucose-induced signals to TOR complex 1, resulting in renal cell pathology in diabetes. *The Journal of Biological Chemistry*.

[24] Kato M, Zhang J, Wang M (2007). MicroRNA-192 in diabetic kidney glomeruli and its function in TGF-*β*-induced collagen expression via inhibition of E-box repressors. *Proceedings of the National Academy of Sciences of the United States of America*.

[25] Kato M, Dang V, Wang M (2013). TGF-*β* induces acetylation of chromatin and of Ets-1 to alleviate repression of miR-192 in diabetic nephropathy. *Science Signaling*.

[26] Putta S, Lanting L, Sun G, Lawson G, Kato M, Natarajan R (2012). Inhibiting microRNA-192 ameliorates renal fibrosis in diabetic nephropathy. *Journal of the American Society of Nephrology*.

[27] Wang B, Herman-Edelstein M, Koh P (2010). E-cadherin expression is regulated by miR-192/215 by a mechanism that is independent of the profibrotic effects of transforming growth factor-*β*. *Diabetes*.

[28] Kato M, Putta S, Wang M (2009). TGF-*β* activates Akt kinase through a microRNA-dependent amplifying circuit targeting PTEN. *Nature Cell Biology*.

[29] Kato M, Wang L, Putta S (2010). Post-transcriptional up-regulation of Tsc-22 by Ybx1, a target of miR-216a, mediates TGF-*β*-induced collagen expression in kidney cells. *The Journal of Biological Chemistry*.

[30] Park JT, Kato M, Yuan H (2013). FOG2 protein down-regulation by transforming growth factor-*β*1-induced MicroRNA-200b/c leads to akt kinase activation and glomerular mesangial hypertrophy related to diabetic nephropathy. *The Journal of Biological Chemistry*.

[31] Bakirtzi K, Hatziapostolou M, Karagiannides I (2011). Neurotensin signaling activates microRNAs-21 and -155 and Akt, promotes tumor growth in mice, and is increased in human colon tumors. *Gastroenterology*.

[32] Darido C, Georgy S, Wilanowski T (2011). Targeting of the tumor suppressor GRHL3 by a miR-21-dependent proto-oncogenic network results in PTEN loss and tumorigenesis. *Cancer Cell*.

[33] Sheedy FJ, Palsson-Mcdermott E, Hennessy EJ (2010). Negative regulation of TLR4 via targeting of the proinflammatory tumor suppressor PDCD4 by the microRNA miR-21. *Nature Immunology*.

[34] Zhong X, Chung ACK, Chen HY (2013). MiR-21 is a key therapeutic target for renal injury in a mouse model of type 2 diabetes. *Diabetologia*.

[35] Fiorentino L, Cavalera M, Mavilio M (2013). Regulation of TIMP3 in diabetic nephropathy: a role for microRNAs. *Acta Diabetologica*.

[36] Basu R, Lee J, Wang Z (2012). Loss of TIMP3 selectively exacerbates diabetic nephropathy. *The American Journal of Physiology: Renal Physiology*.

[37] Wang J, Gao Y, Ma M (2013). Effect of miR-21 on renal fibrosis by regulating MMP-9 and TIMP1 in kk-ay diabetic nephropathy mice. *Cell Biochemistry and Biophysics*.

[38] Wang Q, Wang Y, Minto AW (2008). MicroRNA-377 is up-regulated and can lead to increased fibronectin production in diabetic nephropathy. *The FASEB Journal*.

[39] Beckman JD, Chen C, Nguyen J (2011). Regulation of heme oxygenase-1 protein expression by miR-377 in combination with miR-217. *The Journal of Biological Chemistry*.

[40] Lee SC, Han SH, Li JJ (2009). Induction of heme oxygenase-1 protects against podocyte apoptosis under diabetic conditions. *Kidney International*.

[41] Li H, Zhang L, Wang F (2011). Attenuation of glomerular injury in diabetic mice with tert- butylhydroquinone through nuclear factor erythroid 2-related factor 2-dependent antioxidant gene activation. *American Journal of Nephrology*.

[42] Chen YQ, Wang X, Yao X (2011). MicroRNA-195 promotes apoptosis in mouse podocytes via enhanced caspase activity driven by BCL2 insufficiency. *The American Journal of Nephrology*.

[43] Chen YQ, Wang XX, Yao XM (2012). Abated microRNA-195 expression protected mesangial cells from apoptosis in early diabetic renal injury in mice. *Journal of Nephrology*.

[44] Mortuza R, Feng B, Chakrabarti S (2014). miR-195 regulates SIRT1-mediated changes in diabetic retinopathy. *Diabetologia*.

[45] Bible E (2013). Diabetic nephropathy: Sirt1 attenuates diabetic albuminuria. *Nature Reviews Nephrology*.

[46] Huang K, Huang J, Xie X (2013). Sirt1 resists advanced glycation end products-induced expressions of fibronectin and TGF-beta1 by activating the Nrf2/ARE pathway in glomerular mesangial cells. *Free Radical Biology & Medicine*.

[47] Kitada M, Kume S, Imaizumi N, Koya D (2011). Resveratrol improves oxidative stress and protects against diabetic nephropathy through normalization of Mn-SOD dysfunction in AMPK/SIRT1-independent pathway. *Diabetes*.

[48] Liu R, Zhong Y, Li X (2014). Role of transcription factor acetylation in diabetic kidney disease. *Diabetes*.

[49] Mu J, Pang Q, Guo Y (2013). Functional implications of microRNA-215 in TGF-*β*1-induced phenotypic transition of mesangial cells by targeting CTNNBIP1. *PLoS ONE*.

[50] Sachs N, Sonnenberg A (2013). Cell-matrix adhesion of podocytes in physiology and disease. *Nature Reviews Nephrology*.

[51] Li D, Lu Z, Jia J, Zheng Z, Lin S (2013). Changes in microRNAs associated with podocytic adhesion damage under mechanical stress. *Journal of the Renin-Angiotensin-Aldosterone System*.

[52] Alvarez ML, M. Khosroheidari, E. Eddy, J. Kiefer (2013). Role of microRNA 1207-5P and its host gene, the long non-coding RNA Pvt1, as mediators of extracellular matrix accumulation in the kidney: implications for diabetic nephropathy. *PLoS ONE*.

[53] He F, Peng F, Xia X (2014). MiR-135a promotes renal fibrosis in diabetic nephropathy by regulating TRPC1. *Diabetologia*.

[54] Wang B, Koh P, Winbanks C (2011). MiR-200a prevents renal fibrogenesis through repression of TGF-*β*2 expression. *Diabetes*.

[55] Wei J, Zhang Y, Luo Y (2014). Aldose reductase regulates miR-200a-3p/141-3p to coordinate Keap1-Nrf2, Tgfbeta1/2, and Zeb1/2 signaling in renal mesangial cells and the renal cortex of diabetic mice. *Free Radical Biology & Medicine*.

[56] Wang B, Komers R, Carew R (2012). Suppression of microRNA-29 expression by TGF-*β*1 promotes collagen expression and renal fibrosis. *Journal of the American Society of Nephrology*.

[57] Du B, Ma L, Huang M (2010). High glucose down-regulates miR-29a to increase collagen IV production in HK-2 cells. *FEBS Letters*.

[58] Lin CL, Lee PH, Hsu YC (2014). MicroRNA-29a promotion of nephrin acetylation ameliorates hyperglycemia-induced podocyte dysfunction. *Journal of the American Society of Nephrology*.

[59] Long J, Wang Y, Wang W, Chang BHJ, Danesh FR (2011). MicroRNA-29c is a signature MicroRNA under high glucose conditions that targets sprouty homolog 1, and its in vivo knockdown prevents progression of diabetic nephropathy. *The Journal of Biological Chemistry*.

[60] Zhang Z, Luo X, Ding S (2012). MicroRNA-451 regulates p38 MAPK signaling by targeting of Ywhaz and suppresses the mesangial hypertrophy in early diabetic nephropathy. *The FEBS Letters*.

[61] Yu D, Dos Santos CO, Zhao G (2010). miR-451 protects against erythroid oxidant stress by repressing 14-3-3*ζ*. *Genes and Development*.

[62] Li HP, Zeng XC, Zhang B (2013). miR-451 inhibits cell proliferation in human hepatocellular carcinoma through direct suppression of IKK-beta. *Carcinogenesis*.

[63] Zhang X, Wang X, Zhu H (2010). Synergistic effects of the GATA-4-mediated miR-144/451 cluster in protection against simulated ischemia/reperfusion-induced cardiomyocyte death. *Journal of Molecular and Cellular Cardiology*.

[64] Kato M, Yuan H, Xu Z (2006). Role of the Akt/FoxO3a pathway in TGF-*β*1-mediated mesangial cell dysfunction: a novel mechanism related to diabetic kidney disease. *Journal of the American Society of Nephrology*.

[65] Cheng H, Fan X, Moeckel GW, Harris RC (2011). Podocyte COX-2 exacerbates diabetic nephropathy by increasing podocyte (pro)renin receptor expression. *Journal of the American Society of Nephrology*.

[66] Fu Y, Zhang Y, Wang Z (2010). Regulation of NADPH oxidase activity is associated with miRNA-25-mediated NOX4 expression in experimental diabetic nephropathy. *American Journal of Nephrology*.

[67] Long J, Wang Y, Wang W, Chang BHJ, Danesh FR (2010). Identification of microRNA-93 as a novel regulator of vascular endothelial growth factor in hyperglycemic conditions. *The Journal of Biological Chemistry*.

[68] Wang B, Jha JC, Hagiwara S (2014). Transforming growth factor-*β*1-mediated renal fibrosis is dependent on the regulation of transforming growth factor receptor 1 expression by let-7b. *Kidney International*.

[69] Schaeffer V, Hansen KM, Morris DR, LeBoeuf RC, Abrass CK (2012). RNA-binding protein IGF2BP2/IMP2 is required for laminin-*β*2 mRNA translation and is modulated by glucose concentration. *The American Journal of Physiology—Renal Physiology*.

[70] Ebert MS, Sharp PA (2010). MicroRNA sponges: progress and possibilities. *RNA*.

[71] Stenvang J, Petri A, Lindow M, Obad S, Kauppinen S (2012). Inhibition of microRNA function by antimiR oligonucleotides. *Silence*.

[72] Esau CC (2008). Inhibition of microRNA with antisense oligonucleotides. *Methods*.

[73] Davis S, Propp S, Freier SM (2009). Potent inhibition of microRNA in vivo without degradation. *Nucleic Acids Research*.

[74] Esau CC, Monia BP (2007). Therapeutic potential for microRNAs. *Advanced Drug Delivery Reviews*.

[75] Petersen M, Wengel J (2003). LNA: a versatile tool for therapeutics and genomics. *Trends in Biotechnology*.

[76] Stenvang J, Kauppinen S (2008). MicroRNAs as targets for antisense-based therapeutics. *Expert Opinion on Biological Therapy*.

[77] Braasch DA, Corey DR (2001). Locked nucleic acid (LNA): fine-tuning the recognition of DNA and RNA. *Chemistry and Biology*.

[78] Davis S, Lollo B, Freier S, Esau C (2006). Improved targeting of miRNA with antisense oligonucleotides. *Nucleic Acids Research*.

[79] Ali S, Ahmad A, Banerjee S (2010). Gemcitabine sensitivity can be induced in pancreatic cancer cells through modulation of miR-200 and miR-21 expression by curcumin or its analogue CDF. *Cancer Research*.

[80] Sheth S, Jajoo S, Kaur T (2012). Resveratrol reduces prostate cancer growth and metastasis by inhibiting the Akt/MicroRNA-21 pathway. *PLoS ONE*.

[81] Ebert MS, Neilson JR, Sharp PA (2007). MicroRNA sponges: competitive inhibitors of small RNAs in mammalian cells. *Nature Methods*.

[82] Care A, Catalucci D, Felicetti F (2007). MicroRNA-133 controls cardiac hypertrophy. *Nature Medicine*.

[83] Ma F, Xu S, Liu X (2011). The microRNA miR-29 controls innate and adaptive immune responses to intracellular bacterial infection by targeting interferon-*γ*. *Nature Immunology*.

[84] Valastyan S, Reinhardt F, Benaich N (2009). A pleiotropically acting microRNA, miR-31, inhibits breast cancer metastasis. *Advances in Breast Cancer*.

[85] Shi S, Yu L, Chiu C (2008). Podocyte-selective deletion of dicer induces proteinuria and glomerulosclerosis. *Journal of the American Society of Nephrology*.

[86] Ho J, Kar HN, Rosen S, Dostal A, Gregory RI, Kreidberg JA (2008). Podocyte-specific loss of functional microRNAs leads to rapid glomerular and tubular injury. *Journal of the American Society of Nephrology*.

[87] Deshpande SD, Putta S, Wang M (2013). Transforming growth factor-beta-induced cross talk between p53 and a microRNA in the pathogenesis of diabetic nephropathy. *Diabetes*.

[88] Chau BN, Xin C, Hartner J (2012). MicroRNA-21 promotes fibrosis of the kidney by silencing metabolic pathways. *Science Translational Medicine*.

[89] Henry JC, Azevedo-Pouly ACP, Schmittgen TD (2011). MicroRNA replacement therapy for cancer. *Pharmaceutical Research*.

[90] Ling H, Fabbri M, Calin GA (2013). MicroRNAs and other non-coding RNAs as targets for anticancer drug development. *Nature Reviews Drug Discovery*.

[91] Lan F-F, Wang H, Chen Y-C (2011). *Hsa-let-7g* inhibits proliferation of hepatocellular carcinoma cells by downregulation of *c-Myc* and upregulation of *p16^INK4A^*. *International Journal of Cancer*.

[92] Liu C, Kelnar K, Liu B (2011). The microRNA miR-34a inhibits prostate cancer stem cells and metastasis by directly repressing CD44. *Nature Medicine*.

[93] Su H, Yang J, Xu T (2009). MicroRNA-101, down-regulated in hepatocellular carcinoma, promotes apoptosis and suppresses tumorigenicity. *Cancer Research*.

[94] Xiong Y, Fang J, Yun J (2010). Effects of microrna-29 on apoptosis, tumorigenicity, and prognosis of hepatocellular carcinoma. *Hepatology*.

[95] Liu Z, Sall A, Yang D (2008). MicroRNA: an emerging therapeutic target and intervention tool. *International Journal of Molecular Sciences*.

[96] Zeng Y, Wagner EJ, Cullen BR (2002). Both natural and designed micro RNAs can inhibit the expression of cognate mRNAs when expressed in human cells. *Molecular Cell*.

[97] Grimm D, Streetz KL, Jopling CL (2006). Fatality in mice due to oversaturation of cellular microRNA/short hairpin RNA pathways. *Nature*.

[98] van Balkom BWM, Pisitkun T, Verhaar MC, Knepper MA (2011). Exosomes and the kidney: prospects for diagnosis and therapy of renal diseases. *Kidney International*.

[99] Valadi H, Ekström K, Bossios A, Sjöstrand M, Lee JJ, Lötvall JO (2007). Exosome-mediated transfer of mRNAs and microRNAs is a novel mechanism of genetic exchange between cells. *Nature Cell Biology*.

[100] Chen TS, Lai RC, Lee MM, Choo ABH, Lee CN, Lim SK (2009). Mesenchymal stem cell secretes microparticles enriched in pre-microRNAs. *Nucleic Acids Research*.

[101] Alvarez ML, Khosroheidari M, Kanchi Ravi R, Distefano JK (2012). Comparison of protein, microRNA, and mRNA yields using different methods of urinary exosome isolation for the discovery of kidney disease biomarkers. *Kidney International*.

[102] Barutta F, Tricarico M, Corbelli A (2013). Urinary exosomal microRNAs in incipient diabetic nephropathy. *PLoS One*.

[103] Soubani O, Ali AS, Logna F, Ali S, Philip PA, Sarkar FH (2012). Re-expression of miR-200 by novel approaches regulates the expression of PTEN and MT1-MMP in pancreatic cancer. *Carcinogenesis*.

[104] Li Y, Vandenboom TG, Kong D (2009). Up-regulation of miR-200 and let-7 by natural agents leads to the reversal of epithelial-to-mesenchymal transition in gemcitabine-resistant pancreatic cancer cells. *Cancer Research*.

[105] Wei J, Zhang Y, Luo Y (2013). Aldose reductase regulates miR-200a-3p 141-3p to coordinate Keap1-Nrf2, Tgfbeta1 2 and Zeb1 2 signaling in renal mesangial cells and the renal cortex of diabetic mice. *Free Radical Biology and Medicine*.

[106] Janssen HLA, Reesink HW, Lawitz EJ (2013). Treatment of HCV infection by targeting microRNA. *The New England Journal of Medicine*.

[107] Li Z, Rana TM (2014). Therapeutic targeting of microRNAs: current status and future challenges. *Nature Reviews Drug Discovery*.

